# Identification of *Sarcocystis* spp. in One-humped Camels (*Camelus dromedarius*) from Riyadh and Dammam, Saudi Arabia, via Histological and Phylogenetic Approaches

**DOI:** 10.3390/ani10071108

**Published:** 2020-06-28

**Authors:** Dina M. Metwally, Tahani T. Al-Otaibi, Isra M. Al-Turaiki, Manal F. El-Khadragy, Reem A. Alajmi

**Affiliations:** 1 Department of Zoology, College of Science, King Saud University, Riyadh 11451, KSA; ralajmi@ksu.edu.sa; 2 Department of Parasitology, Faculty of Veterinary Medicine, Zagazig University, Zagazig 44519, Egypt; 3 Department of Biology, Al-Nairiyah University College, University of Hafr Al-Batin, Hafr Al-Batin 31991, Saudi Arabia; ttaalotaibi@gmail.com; 4 Department of Information Technology, College of Computer and Information Sciences, King Saud University, Riyadh 11451, Saudi Arabia; ialturaiki@ksu.edu.sa; 5 Department of Biology, Faculty of Science, Princess Nourah Bint Abdelrahman University, Riyadh 84428, Saudi Arabia; manalelkhadragy@yahoo.com; 6 Department of Zoology and Entomology, Faculty of Science, Helwan University, Cairo 11795, Egypt

**Keywords:** *Sarcocystis* spp., COX1, Camelus dromedarius

## Abstract

**Simple Summary:**

In the present work, we explored the existence of *Sarcocystis* spp. in samples of camels obtained from abattoirs in Riyadh, Saudi Arabia. We examined tissues of the tongue, heart, esophagus, diaphragm, and skeletal muscle by macroscopic assessments, optical microscopy of tissues, optical microscopy of digested sediment, Transmission Electron Microscopy (TEM), and Polymerase chain reaction (PCR) followed by gene sequencing. The results identified *Sarcocystis cameli* (*S. cameli*) and *S. camelicanis*. *Sarcocystis* spp. were detected in Saudi Arabian camels by molecular analysis. *S. levinei* and *S. miescheriana* were most closely related.

**Abstract:**

*Sarcocystis (S.)* spp. are intracellular protozoan parasites that infect birds and animals, resulting in substantial commercial losses. *Sarcocystis* spp. have an indirect life cycle; canines and felines are known to act as final hosts, and numerous domestic and wild animals act as intermediate hosts. The presence of sarcocysts in camel meat may diminish its commercial quality. There is limited knowledge regarding the taxonomy and diagnosis of *Sarcocystis* spp. that infect camels in Saudi Arabia. In this study, transmission electron microscopy (TEM) revealed *S. cameli* and *S. camelicanis* (*camelicanis*) in *Camelus (C.) dromedarius*. This is the first report of *S. camelicanis* in Saudi Arabia and is considered a significant finding. Based on cytochrome c oxidase subunit I gene (COX1) sequences, two samples of *Sarcocystis* spp. isolated from *C. dromedarius* in Riyadh and Dammam were grouped with *S. levinei* hosted by *Bubalus bubalis* in India, *S. rangi* hosted by *Rangifer tarandus* in Norway, *S. miescheriana* hosted by *Sus scrofa* in Italy and *S. fayeri* hosted by *Equus caballus* in Canada. The sequences obtained in this study have been deposited in GenBank.

## 1. Introduction

*Sarcocystis (S.)* is a genus of intracellular coccidian parasites that was first identified in 1843 [1]. More than 200 species have been identified and were demonstrated to infect a wide range of domestic and wild animals, resulting in significant losses in farm animals worldwide [2,3]. *Sarcocystis* spp. accomplish their life cycles in two hosts, a final and an intermediate host, and are known to have a high level of host specificity with regard to their intermediate hosts as opposed to their final hosts [4]. The asexual stages of *Sarcocystis* development occur in intermediate hosts (herbivorous animals, such as sheep, cattle and camels, and primates, such as humans, poikilothermic animals and birds), where sarcocysts generally become visible in skeletal muscles and in the striated muscles of the heart, diaphragm and esophagus [5,6] but are rarely found in the smooth muscle of the intestine and the central nervous system [4,5,6,7]. The final hosts (e.g., canids, felids, marsupials and primates) become infected by eating intermediate host tissue infected with mature sarcocysts. Next, a stage of sexual reproduction occurs in the final host, after which oocysts/sporocysts are expelled into the environment through feces to be eaten by appropriate intermediate hosts.

The one-humped camel *Camelus dromedarius* is broadly distributed in the hot, arid regions of the Middle East, South Asia, Africa, the Canary Islands, and Central Australia. Compared to beef, lamb, and ostrich meat, camel meat is favored in several countries due to its reduced fat and cholesterol contents [8,9]. However, the presence of sarcocysts in camel meat may decrease its value for human utilization because camels act as intermediate hosts for at least two *Sarcocystis* spp.; furthermore, such infection is a general phenomenon in the one-humped camel due to its worldwide distribution [10,11,12]. The presence of sarcocysts in the musculature decreases the economic value of the musculature, especially if macroscopic cysts are detected, as their presence leads to condemning the product for consumption [13,14]. Microscopic cysts, on the other hand, may cause serious pathological conditions in infected animals, especially in the case of acute forms, and result in heavy production losses [15,16,17].

Infected camels generally exhibit subclinical infections, although *Sarcocystis* spp. can produce extensive pathology or death in these animals [12,18].

The diagnosis of acute sarcocystosis in camels is an onerous task because of the absence of an industrial standard indicator test, asymptomatic features of the infection, and the presence of microscopic sarcocysts. Muscle squash, pepsin digestion, trypsin digestion and histopathological examination have been used for the analysis of microscopic sarcocysts in camels [11,12,18,19]. Ultrastructural analysis of the cyst wall is quite useful in the identification of sarcocysts in camels. Transmission electron microscopy (TEM) has demonstrated that *S. cameli* has a primary cyst wall that appears as a thin wall with finger-like villar protrusions and rows of knob-like projections. *S. ippeni* was defined in Egypt [20], wherein the authors of that study observed cyst walls harboring cone-like villar protrusions. *S. camelicanis* was also defined in Egypt [19], wherein the authors found that the primary cyst wall appeared as a thick layer and had finger-like protrusions with a blunt apex. Furthermore, another study defined *S. miescheri* in Egypt [10], in which the primary cyst wall appeared as a thick electron-dense layer with spine-like protrusions.

Although molecular analysis might be an alternative technique for the identification of *Sarcocystis* spp., there is a limitation of such data for camels. The first molecular identification of *S. cameli* sarcocysts was performed in camels in Iran, wherein the 18S rRNA gene fragment was amplified from bradyzoite DNA using conventional polymerase chain reaction (PCR), followed by sequencing (GenBank: GU074011.1) and restriction fragment length polymorphism (RFLP) analysis [21,22]. However, the RFLP technique was found to be more expensive than DNA sequencing or electron microscopy techniques. In another study, the 18S rRNA gene was amplified from microscopic sarcocysts in camels, although no phylogenetic analysis was reported [12]. Therefore, in the present study, a molecular marker, cytochrome oxidase subunit I (COX1), was used to identify different *Sarcocystis* spp. infecting camels from Riyadh and Dammam, Saudi Arabia.

## 2. Materials and Methods

### 2.1. Sample Collection

Between February and October 2018, veterinarians collected samples of tissues (diaphragm, skeletal muscle, cardiac muscle, tongue, and esophagus) during postmortem investigations of slaughtered animals in the West Abattoir and Dammam Slaughterhouses in Riyadh and Dammam, Saudi Arabia, respectively. Tissue samples were collected from 37 (27 in Riyadh and 10 in Dammam) male camels aged >5 years and transported to the laboratory in boxes containing ice packs. This study (IRB number: KSU-SE-18-33) secured the approval of the Institutional Committee of Postgraduate Studies and Research at King Saud University (Saudi Arabia).

### 2.2. Macroscopic Examination

On the same day as the tissues were collected, a macroscopic analysis was performed. In this process, a scalpel was used to make as many as five transverse incisions on organs, such as the heart and the tongue, for the purpose of revealing the cysts (macroscopic). A macroscopic analysis of the entire esophagus was also performed to examine the external and internal walls; the lumen was exposed after longitudinal sectioning [23].

### 2.3. Microscopic Examination

Microscopic evaluation of cysts was performed using the squashing method [24]. Distinct fragments of each tissue with a thickness of approximately 5 mm were squashed robustly from both slides, after which an optical microscope was used to analyze the specimen. For all tissues, the procedure was performed in triplicate. *Sarcocystis* spp. were also examined by light microscopy and DNA analyses.

### 2.4. Digestion Method

For all tissues, approximately 20 g were minced before digestion for a period of 30 min in 100 mL at a temperature of 40 °C; the digestion solution comprised pepsin (1.3 g), HCl (3.5 mL) and NaCl (2.5 g) in distilled water (500 mL) [25]. After digestion, the mixture was centrifuged at 3500× *g* for 3 min, followed by Giemsa staining and optical microscopy evaluation [26].

### 2.5. Histopathological Examination

Small specimens of muscles and organs were collected, fixed in 10% neutral buffered formalin, serially dehydrated in increasing concentrations of ethanol (30%, 70% and 95% absolute) and embedded in paraffin. Three 5-µm-thick sections of each of the abovementioned organs and muscles were prepared, stained with hematoxylin and eosin and examined under an ECLIPSE NI-4 (Nikon, Tokyo, Japan) [27].

### 2.6. Transmission Electron Microscopy (TEM)

Six *Sarcocystis* spp. cysts embedded in the tissues were collected from organs, fixed in 0.1 M sodium cacodylate buffer (pH 7.4) supplemented with 3% glutaraldehyde solution for 4 h at 4 °C and stored at 4 °C until processing. After fixation, the samples were washed in 0.1 M sodium cacodylate buffer, fixed with 2% osmium tetroxide for 24 h and rewashed four to five times in the buffer (10–15 min each) [19]. The samples were serially dehydrated in increasing concentrations of acetone (30%, 40%, 50%, 70%, 90% and 100%) and blocked with buffer supplemented with 1% phosphotungstic acid and 1% uranyl acetate. Next, the 100% acetone solution was replaced with Polybed resin, followed by paraffin embedding and polymerization in an oven at 60 °C [28]. Moderately thin sections were prepared to observe *Sarcocystis* spp. cysts under a microscope (CX31, Olympus Corporation, Tokyo, Japan). Ultrathin sections were stained with uranyl acetate and lead citrate and examined using a JEM-1400 transmission electron microscope (JEOL, Tokyo, Japan) at 80 kV.

### 2.7. Molecular Analysis

#### 2.7.1. DNA Extraction and PCR Amplification

From all the microscopic *Sarcocystis* isolates, six isolates were selected and washed five times in distilled water (sterile). gDNA from tissue was extracted using a DNA Mini Kit (QIAGEN GmbH, Hilden, Germany) (Cat. No. 51304) according to the manufacturer’s instructions. Amplification of the COX1 gene was performed using the primer pairs mentioned in Table 1 [28,29] and a thermocycler (Veriti^®^ 96-well Thermal Cycler, Model 9902, Biosystem). This procedure was conducted in a mixture (20 µL) containing 4 µL of master mix (5×), 12 µL of RNase-free water and DNA template (2 µL). The PCR program consisted of denaturation at 95 °C for 5 min, followed by 40 cycles of denaturation for 45 s at 95 °C, annealing for 45 s at 54 °C and extension for 10 min at 72 °C. The PCR products were analyzed by 1.5% agarose gel electrophoresis.

#### 2.7.2. DNA Sequencing

The aforementioned PCR products were first purified and then sequenced in the reverse and forward directions (only) using a Genetic Analyzer at the Central Laboratory of King Saud University.

The sequences were analyzed using Geneious Prime Build [31]. All sequences were truncated slightly using the error probability method with a limit of 0.05 at both ends. A Basic Local Alignment Search Tool (BLAST) search was performed to identify related sequences. Multiple sequence alignments were generated using CLUSTAL Omega [32]. The maximum likelihood trees were constructed using PhyML 3.3 with 100 bootstraps [33].

### 2.8. Statistical Analysis

Statistical analysis was performed using the Statistical Package for Social Sciences (SPSS) software (version 17, SPSS, Inc., Chicago, IL, USA). The lengths and widths of at least five cysts from each organ (diaphragm, skeletal muscle, cardiac muscle, tongue, and esophagus) were determined by light microscopy and expressed as the mean sizes and amplitudes of variation.

## 3. Results

### 3.1. Macroscopic Examination

No macroscopic cysts were detected by the naked eye during the inspection of carcasses or collected samples.

### 3.2. Microscopic Examination

A compression technique with a light microscope was used for microscopic examination, in which 15 of 37 slaughtered camels (40.54%) were found to be positive for microscopic cysts. In particular, the numbers of positive cases for diaphragm, skeletal muscle, cardiac muscle, tongue, and esophagus samples were 14/37 (37.83%), 10/37 (27.01%), 9/37 (24.32%), 8/37 (21.62%), and 3/37 (8.10%), respectively. Thin-walled sarcocysts and thick-walled sarcocysts were observed. In contrast to the tissue squash method, the pepsin–hydrochloric acid digestion technique revealed a greater number of animals that were positive.

#### 3.2.1. Thin-Walled Microscopic Sarcocysts

**Figure 1:**
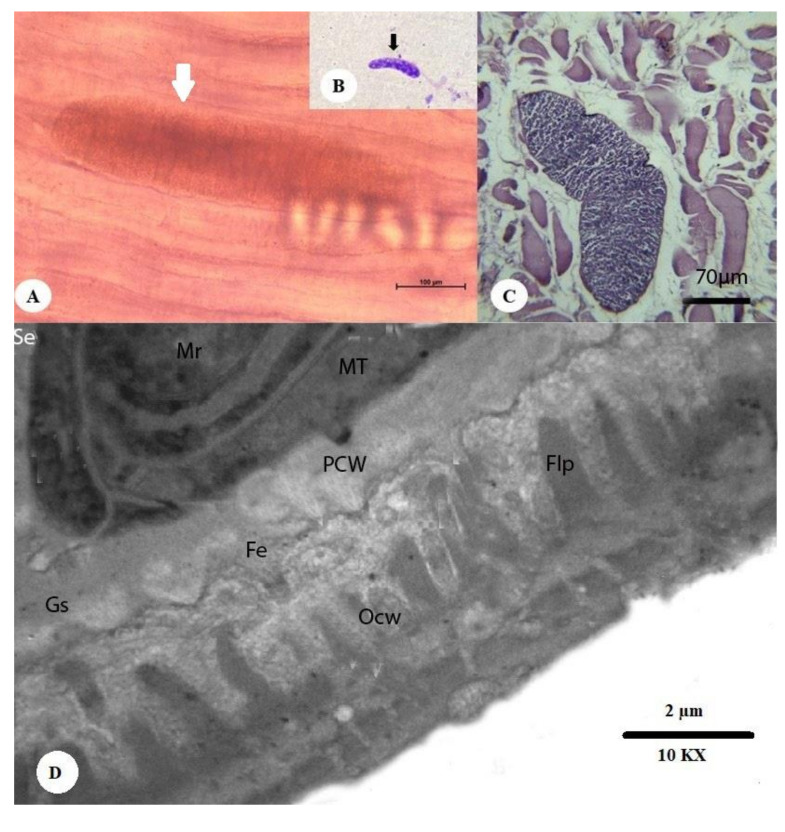
Morphology of a sarcocyst of *S. cameli* from the esophageal muscles of a camel (*Camelus dromedarius*). (**A**) Microscopic thin-walled *S. cameli* (white arrow) (bar = 100 µm). (**B**) Cysts stained with Giemsa after pepsin–hydrochloric acid digestion (black arrow). (**C**) Histopathological section of camel esophageal muscles showing the thin cyst wall (CW) of *S. cameli* (H&E) (bar = 70 µm). The thin and smooth cyst wall and the clearly visible septum are shown. (**D**) The cyst wall in detail, containing an outer cyst wall (Ocw), a primary cyst wall (Pcw), ground substance (Gs), septae (Se), merozoites (Mr), fibrillar elements (Fe), metrocytes (Mt), and finger-like process (Flp) (10,000×).

Unstained sarcocysts were found to have thin walls, to be elongated and spindle-like in shape, and to be present within the muscle fibers. The sarcocysts measured 197.9–405.6 µm in length (mean 301.75 µm) and 57.3–125.6 µm in breadth (mean 91.45 µm) (Figure 1A). Stained sarcocysts are shown in Figure 1B. Histopathological sections revealed that the cysts measured 83.50–135.50 µm in length (mean 109.5 µm). The cyst wall consisted of two layers, an outer striated layer and an inner homogenous layer. The cystic cavity was divided into several compartments by fine trabeculae originating from the cyst wall (Figure 1C). Ultrastructural analysis of the cyst wall showed that *S. cameli* sarcocysts (shown in Figure 1D) have an outer cyst wall (Ocw) that is in close contact with the cyst wall, ground substance, metrocytes, and bradyzoites or merozoites. The primary cyst wall (Pcw) displayed irregular folded nonbranched finger-like villar protrusions with fibrillar elements originating from the ground substance at a distance of 0.54 µm below the primary cyst wall and protruding into it. The villar protrusions measured 0.93 µm in length and 0.77 µm in width at the base and 0.35 µm at the apex. The distance between each villar protrusion measured 1.7 µm. The finger-like process (Flp) was generally surrounded by numerous scattered host cell mitochondria. The ground substance was located directly under the primary cyst wall with a diameter of 1.27–1.7 µm (mean: 1.48 µm). The space between the primary cyst wall (Pcw) and the contents of the cyst was primarily fine, showing dense homogenous granules and fibrillar elements folding into the primary cyst wall. The ground substance extended into the interior of the cyst, forming a thin septum separating the entire cyst into compartments enclosing the metrocytes and bradyzoites or merozoites.

#### 3.2.2. Thick-walled Microscopic Sarcocysts

**Figure 2:**
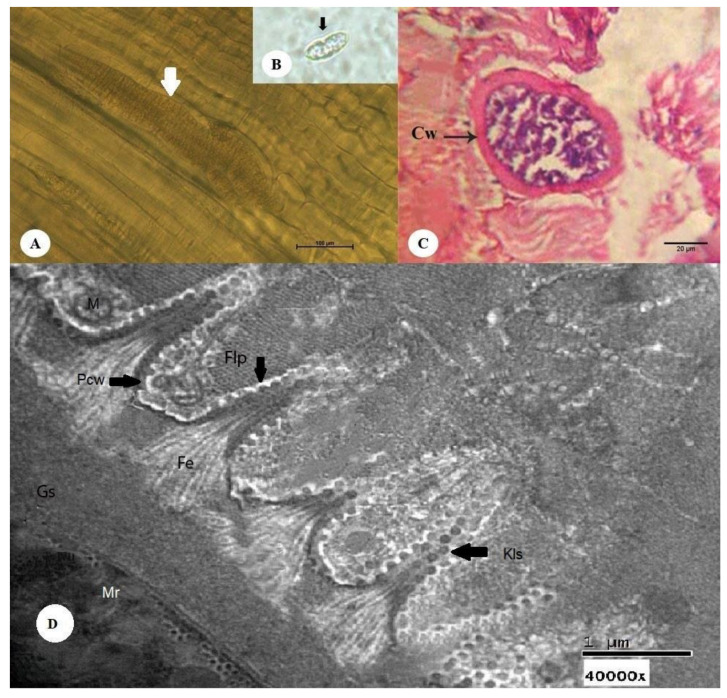
Morphology of a sarcocyst of *S. camelicanis* from the skeletal muscles of a camel (*Camelus dromedarius*). (**A**) Microscopic thick-walled *S. camelicanis* (white arrow) (bar = 100 µm). (**B**) Cysts after pepsin–hydrochloric acid digestion (small black arrow). (**C**) A histopathological section of the skeletal muscles of a camel shows the thick cyst wall (Cw) (black arrow) of *S. camelicanis* and a clearly visible septum (H&E; bar = 20 µm). (**D**) A *Sarcocystis camelicanis* thick-walled cyst showing the primary cyst wall (Pcw) (black arrow), ground substance (Gs), fibrillar elements (Fe), finger-like process (Flp), knoblike structures (Kls) (black arrow), Mitochondria (M), and merozoites (Mr) (40,000×).

The sarcocysts appeared fusiform or spindle-shaped, measuring 151–449 µm in length (mean 300 µm) and 65–140 µm in breadth (mean 102.5 µm) (Figure 2A). Cysts after pepsin–hydrochloric acid digestion are depicted in Figure 2B. Histopathological sections demonstrated that the cysts had a thick wall composed of two layers: an outer striated layer and an inner smooth layer. The cyst cavity was divided into compartments by a narrow septum originating from the wall in the periphery of the cyst (Figure 2C). Ultrastructural analysis (Figure 1D) of the cyst wall revealed an outer cyst wall connected to the primary cyst wall (Pcw), ground substance (Gs), metrocytes, and bradyzoites for *S. camelicanis*.

The primary cyst wall (Pcw) is a thick, dense layer adjacent to the Gs. Fibrillar elements (Fe) originating from the Gs at 0.60 µm below the Pcw aggregated towards the Pcw and embedded into it, forming finger-like protrusions (Flp). The cyst measured 2.15–2.91 µm (mean 2.53 µm) in length and 0.52–0.60 µm (mean 0.56 µm) in breadth. Each Flp carried characteristic multiple numerous knob-like structures (Kls), which were spherical in shape with a diameter of 0.10–0.14 µm (mean 0.12 µm); their number varied from 19 to 25 on each protrusion. The distance between each Flp was 0.71–0.89 µm (mean 0.80 µm), and they were generally surrounded by numerous dispersed host cell mitochondria (M). The Gs was found to be 1.25–1.60 µm (mean 1.42 µm) below the Pcw and in between the Pcw and metrocytes. It appeared as a homogeneous substance and extended to the interior of the cyst by a septum separating the entire cyst into a number of compartments enclosing the metrocytes, merozoites and other structures.

### 3.3. Genetic Characteristics

Genomic DNA extracted from four *Sarcocystis* spp. isolates, D7S (thin-walled cyst), D10S (thick-walled cyst), R10C (thick-walled cyst) and R16C (thin-walled cyst), were used for the amplification of 1000-bp COX1 sequences.

The first two were isolated from the skeletal muscle of a Dammam camel, and the last two were isolated from the cardiac muscle of a Riyadh camel. These sequences were 692, 513, 194, and 205 nucleotides in length, respectively, and shared a pairwise identity of 63.1%. All sequences obtained in this study have been deposited in GenBank under accession numbers MK948444 (D7S), MK948443 (D10S), MK948442 (R10C), and MK948441 (R16C).

BLAST results showed that the D7S sequence had similarity to *S. levinei* hosted by *Bubalus bubalis* (heart tissue) in India, accession numbers MH255774–MH255777, with 86.5% pairwise identity and 89.6% coverage. The D7S sequence also displayed similarity to *S. rangi* hosted by *Rangifer tarandus* in Norway, accession numbers KC209662–KC209666, with 85.2% pairwise identity and 87.57% coverage.

**Figure 3:**
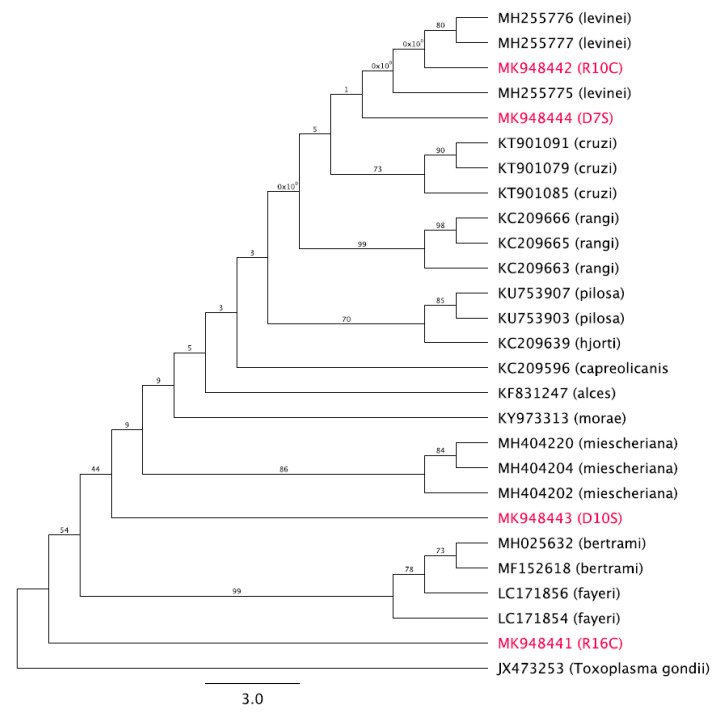
Genetic relationships of our samples isolated from camels in Saudi Arabia (highlighted in pink) with other *Sarcocystis* spp. retrieved from GenBank based on the COX1 region.

Furthermore, the D10S sequence exhibited similarity to *S. miescheriana*, accession number MH404202, hosted by *Sus scrofa* in Italy, with 80.7% pairwise identity and 43% coverage. The D10S sequence was also similar to *S. fayeri*, accession number LC171854, hosted by *Equus caballus* in Canada, with 79.1% pairwise identity and 43% coverage. The phylogenetic tree with bootstrap proportions is illustrated in Figure 3. *Toxoplasma gondii* (JX473253) was used as an outgroup. For R10C and R16C, ‘194 and 205′ are very short sequences inappropriate for analysis. The tree shows that MK948444 (D7S) and MK948442 (R10C) are placed in a clade with *S. levinei.* MK948443 (D10S) is grouped with *Sarcocystis miescheriana.* MK948441 (R16C) is grouped with *Sarcocystis fayeri.*

## 4. Discussion

To establish the occurrence of *Sarcocystis* spp., this study examined the esophagus, tongue, diaphragm, skeletal muscle, and heart tissue from 37 camel specimens (27 from Riyadh and 10 from Dammam). The results showed that the overall prevalence rates of *Sarcocystis* spp. were 40.74% (11/27) in Riyadh specimens and 40% (4/10) in Dammam specimens.

In the present study, macroscopic cysts were not detected. However, macroscopic cysts have been reported to be less common in some esophageal samples [34,35]. In this study, the squashing and pepsin-hydrochloric acid digestion methods accompanied by light microscopy were used, wherein the technique of pepsin-hydrochloric acid digestion revealed a greater number of positive animals. Cysts from esophageal and skeletal muscle samples appeared in a cylindrical and spindle-like shape.

Cysts were found to have thin and smooth or thick walls divided internally into several compartments by trabeculae. Thin-walled cysts have been identified in the esophagus and diaphragm of camels slaughtered at Al-Ahsa Abattoir, Saudi Arabia [36]; in the esophagus, diaphragm, heart, shoulder, and masseter muscles of one-humped camels slaughtered in southern Ethiopia [37]; in the esophagus, diaphragm, heart, skeletal muscles, and tongue of one-humped camels slaughtered in Iran [18], and in the esophagus of one-humped camels slaughtered in Egypt [38].

Thick-walled cysts were found in the esophagus of one-humped camels slaughtered in Egypt [10]; in the esophagus, diaphragm, heart, skeletal muscles, and tongue of one-humped camels slaughtered in Egypt [19]; in the esophagus of one-humped camels slaughtered in Egypt [39], and in the esophagus, skeletal muscles, and tongue of one-humped camels slaughtered in Egypt [40]. Similarly, both thin- and thick-walled *Sarcocystis* cysts have been suggested to be at different stages in the same parasite and have been termed *S. cameli* [41]. In addition, thick-walled cysts were found repeatedly and termed *S. cameli*, whereas thin-walled cysts were unnamed [18,25,36].

*Sarcocystis* spp. isolated from *C. dromedarius* from Dammam and Riyadh grouped alongside *S. levinei* hosted by *B. bubalis* (heart tissue) in India (accession numbers MH255774–MH255777) [42], *S. rangi* hosted by *R. tarandus* in Norway (accession numbers KC209662–KC209666) [43], *S. miescheriana* hosted by *S. scrofa* in Italy (accession number MH404202) [44], and *S. fayeri* hosted by *E. caballus* in Canada (accession number LC171854) [45].

In the present study, four partial sequences of the COX1 gene were analyzed. Two samples, R10C (thick-walled cyst) and D7S (thin-walled cyst), isolated from *C. dromedarius* in Riyadh and Dammam, Saudi Arabia, respectively, were identified by histological and TEM approaches as *Sarcocystis* spp. The phylogenetic tree shows that these two samples are related to *S. mansoni* hosted by South American camelids, *S. levinei* hosted by water buffaloes, and *S. miescheriana* hosted by pigs, suggesting that camels are receptors of *Sarcocystis* spp. from other intermediate hosts. 

The main limitation of this study lies in the other two samples, D10S and R16C. These two samples were identified by histological and TEM approaches as *Sarcocystis* spp. (D10S, thick-walled cyst; and R16C, thin-walled cyst). However, in the phylogenetic tree, they were not grouped in a clade with R10C and D7S. Through a deeper analysis of the raw data, it was found that the samples were of lower quality than R10C and D7S. This low quality affected the process of phylogenetic tree construction.

## 5. Conclusions

In this study, our results highlight the necessity to reinforce a more in-depth phylogenetic analysis and identification of *Sarcocystis* spp. with more taxa and different molecular markers. For instance, amplification of the 18S rRNA gene should be performed to further distinguish among the closely related *Sarcocystis* spp.

## Figures and Tables

**Table 1:** List of primers used for the amplification of cytochrome c oxidase subunit I (COX1) gene in *Sarcocystis* spp.

Gene	Primers	Sequences	References
COX1	SF1	5′-ATG GCG TAC AAC AAT CAT AAA GAA-3′	[28,29,30]
	SR9	5′-ATA TCC ATA CCR CCA TTG CCC AT-3′	
